# Aflatoxin B1 Control by Various *Pseudomonas* Isolates

**DOI:** 10.3390/toxins16080367

**Published:** 2024-08-20

**Authors:** Dóra Anna Papp, Sándor Kocsubé, Zoltán Farkas, András Szekeres, Csaba Vágvölgyi, Zsuzsanna Hamari, Mónika Varga

**Affiliations:** 1 Department of Biotechnology and Microbiology, Institute of Science and Informatics, University of Szeged, 6726 Szeged, Hungary; 2 HCEMM-USZ Functional Cell Biology and Immunology Advanced Core Facility, University of Szeged, 6726 Szeged, Hungary; 3 Synthetic and Systems Biology Unit, Institute of Biochemistry, Biological Research Centre, Eötvös Loránd Research Network, 6726 Szeged, Hungary

**Keywords:** aflatoxin, *Aspergillus flavus*, *Pseudomonas*, biosynthesis, degradation, antagonism, biocontrol

## Abstract

The climate-change-coupled fungal burden in crop management and the need to reduce chemical pesticide usage highlight the importance of finding sustainable ways to control *Aspergillus flavus*. This study examines the effectiveness of 50 *Pseudomonas* isolates obtained from corn rhizospheres against *A. flavus* in both solid and liquid co-cultures. The presence and quantity of aflatoxin B1 (AFB1) and AFB1-related compounds were determined using high-performance liquid chromatography–high resolution mass spectrometry analysis. Various enzymatic- or non-enzymatic mechanisms are proposed to interpret the decrease in AFB1 production, accompanied by the accumulation of biosynthetic intermediates (11-hydroxy-O-methylsterigmatocystin, aspertoxin, 11-hydroxyaspertoxin) or degradation products (the compounds C_16_H_10_O_6_, C_16_H_14_O_5_, C_18_H_16_O_7_, and C_19_H_16_O_8_). Our finding implies the upregulation or enhanced activity of fungal oxidoreductases and laccases in response to bacterial bioactive compound(s). Furthermore, non-enzymatic reactions resulted in the formation of additional degradation products due to acid accumulation in the fermented broth. Three isolates completely inhibited AFB1 or any AFB1-related compounds without significantly affecting fungal growth. These bacterial isolates supposedly block the entire pathway for AFB1 production in the fungus during interaction. Apart from identifying effective *Pseudomonas* isolates as potential biocontrol agents, this work lays the foundation for exploring new bacterial bioactive compounds.

## 1. Introduction

The saprophytic fungus *Aspergillus flavus* causes significant economic losses not only by reducing crop quality but also by contaminating seeds with aflatoxins. Among them, aflatoxin B1 is the most carcinogenic, mutagenic, and immunosuppressive. It has been classified as a class I human carcinogen by the International Agency for Research on Cancer [1]. Feed contaminated with AFB1 has a negative impact on the growth rate, immunity to diseases, and reproductive capability of livestock [2]. In mammals, AFB1 can accumulate in muscles and internal organs or be excreted through milk after conversion to a toxic metabolite [3]. Poultry, especially turkeys, have been shown to be highly sensitive to AFB1, which causes pathological changes, mainly in the liver [4,5]. Therefore, strict regulations have been implemented in many countries to control AF contamination in food (0.5–15 µg/kg) and feed (0.5–300 µg/kg) [6] to decrease the public health risk. *A. flavus* primarily infects plants that are exposed to environmental stresses, including drought and heat stress, via damages caused by insects. It predominantly affects regions in Africa, Asia, and South America, characterized by warm and humid climates, although it is also found in specific areas of North America and Europe. Unfortunately, multiple factors are expected to facilitate the spreading of *A. flavus* and other molds into previously unaffected regions. These factors are dominantly associated with climate change, including increased drought stress, changes in crop phenology, and increased pest reproduction rates.

To control *A. flavus* infection, various pre-harvest strategies have been implemented, including field management practices, breeding of resistant crop varieties, the application of synthetic chemicals, and the use of competitive atoxigenic strains of *A. flavus* [7]. Besides maintaining proper storage conditions (temperature, humidity), a range of physical (adsorption, shorting, dehulling, UV and gamma irradiation) and chemical (organic and inorganic acids, gases) methods are widely employed as post-harvest management strategies [8,9]. Numerous studies have focused on identifying biocontrol agents, including fungi, yeast, and bacteria, to prevent *A. flavus* infection and control aflatoxin production (reviewed in Ren et al., 2020 [10] and references therein). Biocontrol agents might compete for nutrients and space and/or inhibit the biosynthetic pathway by releasing various enzymes and/or diffusible or volatile metabolites [11,12,13,14]. Post-harvest biocontrol strategies include the elimination of the toxin by microbial binding [15] or biodegradation [16]. It is worth nothing that microbial binding is reversible depending on the interacting strain, treatment, and environmental conditions [17]. Regarding biodegradation, the process does not necessarily result in less toxic compounds, and therefore, it is crucial to evaluate the structure, quantity, and toxicity of the degradation end-products. Modification of the furofuran or lactone rings has been shown to result in the loss of mutagenic activity or a reduction in toxicity [18]. Extracellular enzymes, such as extracellular laccases, peroxidases, lactonases, and reductases, are reported to be involved in the degradation process [18]. However, only a few enzymes, e.g., aflatoxin-detoxifizyme [19], F420 H2-dependent reductase [20], and Mn peroxidase [21], have been identified and characterized so far.

In line with the proposed role of the plant microbiome as an antagonist of plant pathogen organisms, different species of *Pseudomonas* isolated from almond fruits [22], maize rhizosphere [23], rice grains [24], and wheat soil [25] have been identified as effective biocontrol agents against *A. flavus*, inhibiting fungal growth and/or toxin biosynthesis. *P. fluorescens*, *P. putida,* and *P. chlororaphis* [22,23] have been shown to reduce *A. flavus* growth by 80–96%, while *P. syringae* [22] and *P. protegens* [24] reduce mycelial growth by 50–65%. Certain *Pseudomonas* sp. such as *P. aeruginosa* [26,27], *P. anguilliseptica* [28], *P. fluorescens* [28,29], *P. protegens* [24], and *P. putida* [30] have been reported to completely or partially degrade AFB1. According to Singh and Mehta, a lipase is responsible for the degradation of AFB1 by *P. putida* [31]. Yao et al. reported a non-enzymatic transformation of AFB1 by *P. geniculate*. They found that 1,2- or 1,1-dimethylhydrazine produced by the bacteria was capable of opening the lactone ring of the toxin [25].

Many studies have focused on the antagonistic interactions between various *Pseudomonas* species and *A. flavus*. These studies have led to the identification of numerous *Pseudomonas* strains with potential biocontrol capabilities. It is essential to identify additional potential biocontrol species in order to achieve sustainable biocontrol that is capable of withstanding diverse climatic conditions and different soil compositions and microbial ecosystems with great diversity at the site of applications. These species should be compatible for use in conjunction with other biocontrol agents, ensuring a comprehensive and adaptable strategy for managing *A. flavus* infections.

## 2. Results and Discussion

### 2.1. Isolation and Identification of Pseudomonas Isolates

**Figure 1:**
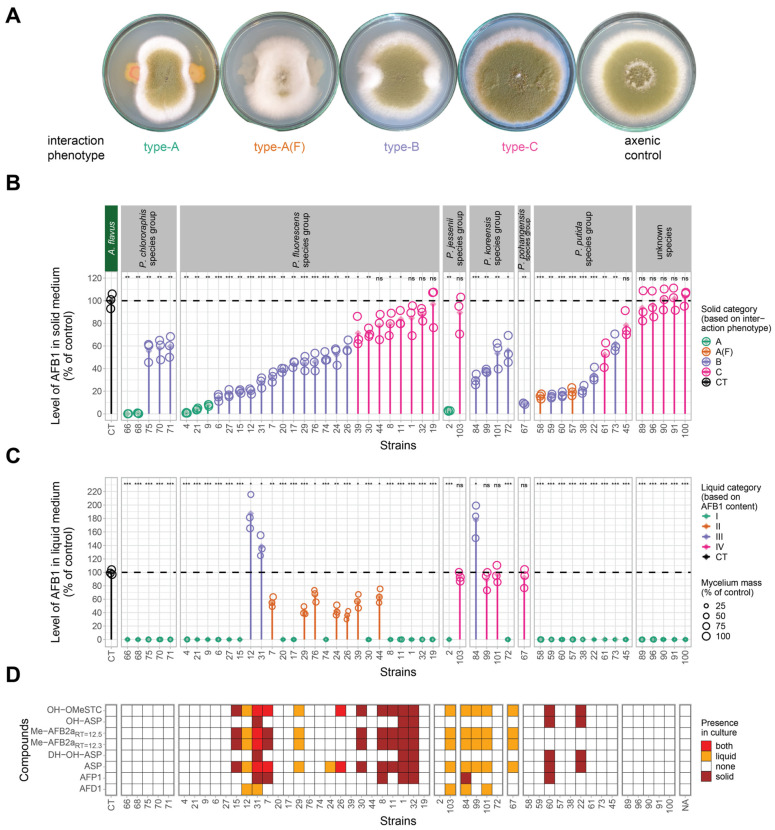
Monitoring of interaction phenotypes and production of AFB1 and AFB1-related compounds in solid and liquid co-cultures of *A. flavus* with 50 *Pseudomonas* isolates. (**A**): The morphology of interaction phenotypes in solid co-cultures. Axenic control shows the morphology of *A. flavus* mono-culture. (**B**): Graphical presentation of AFB1 content of agar disks cut from the center of fungal colonies of solid co-cultures in % of the AFB1 content of the axenic *A. flavus* control. Colors indicate the type of interaction presented in Figure 1A. CT on the *X*-axis denotes the axenic control. The numbers on the *X*-axis confer to the collection names of the co-cultured *Pseudomonas* isolates. A dashed line marks the reference value of the axenic control (100%). Raw data points of the three biological replicates (empty circles) and mean values (full diamonds) are shown. Significant differences (Student’s *t*-test) are marked with asterisks (* *p* < 0.05; ** *p* < 0.01; *** *p* < 0.001; ns: non-significant). For raw data, see Table S1. (**C**): Graphical presentation of the AFB1 content of the fermented broth of liquid co-cultures in % of the AFB1 content of the axenic *A. flavus* control. Color and figure codes are shown in the figure legend. The colors indicate the type of liquid interactions (explained in the main text). The size of the circles indicates the dry weight of the fungal biomass derived from liquid co-cultures in % of that of the axenic control. Explanation of X-axis, dashed line and significant differences is the same as in Figure 1A. The raw data points of the three biological replicates (empty circles) and mean values (full diamonds) are shown. For raw data, see Table S1. (**D**): Heat map presentation of the appearance of AFB1-related compounds in the solid and liquid co-cultures and in the axenic control. Color codes are denoted in the legend. The detected compounds were OH-OMeSTC (11-hydroxy-O-methylsterigmatocystin), OH-ASP (11-hydroxyaspertoxin), Me-AFB2a_RT=12.5_, and Me-AFB2a_RT=12.3_ (two isomers of methoxy-aflatoxin B2a), ASP (aspertoxin), AFP1 (aflatoxin P1), and AFD1 (aflatoxin D1). For raw data, see Table S2.

A total of 50 *Pseudomonas* isolates were obtained and purified from corn rhizosphere samples at three different times of the growing season using a *Pseudomonas*-selective medium [32]. The sequence analysis of the 16S rRNA gene confirmed that the isolates belong to the *Pseudomonas* genus. All isolates were further characterized by sequence analysis of the rpoD gene (encodes the sigma 70 factor of RNA polymerase) [33]. According to the phylogenetic classification of Girard et al. [34], 46% of the isolates belonged to the *P. fluorescens* species group, while 18%, 10%, 8%, 4%, 2%, and 2% of the isolates belonged to the *P. putida*, *P. chlororaphis*, *P. koreensis*, *P. jessenii*, *P. gessardii,* and *P. pohangensis* species groups, respectively (Figure 1B). Five isolates (10%) were identical according to the 16S rRNA and *rpoD* gene sequences; however, they could not be assigned to any known *Pseudomonas* species or species group.

### 2.2. Effect of Pseudomonas Isolates on Growth and AFB1 Production of A. flavus on Solid PD Medium (PDA)

To assess the antagonistic activity of isolates against *A. flavus*, co-cultures were set up with *A. flavus* on a PDA medium for 7 days at 25 °C. Based on the fungal morphology, four types of interactions were distinguished (Figure 1A).

In type-A and -A(F) interactions, the fungal growth was incompletely inhibited around the bacterial colony. Furthermore, in type-A(F) (F indicates fluffy phenotype), the fungal conidiogenesis was decreased, whereas the aerial hypha formation was increased, through the entire fungal colony. In type-B interaction, the fungal growth was only slightly inhibited, as the fungus was able to overgrow on the bacterial colony. Notably, the conidiogenesis was locally decreased in the mycelia covering the bacterial colony, whereas aerial hypha formation was increased. In type-C interaction, the fungal growth was not affected by the bacteria. Mycelia could overgrow the bacterial colony, as in type-B interactions; however, conidiogenesis was evenly wild-type-like through the entire fungal colony. The lack of complete inhibition of *A. flavus* growth on the solid medium is in line with the reported antagonistic properties of different *Pseudomonas* isolates [22,23].

Given the capacity of bacteria to influence both fungal growth and toxin accumulation, the AFB1 content of the co-cultured fungal colonies was determined and compared to that of a non-co-cultured, sole colony (axenic colony). It is important to highlight that a toxin measurement was performed on samples taken from the central region of the colonies via removal of a circular agar disk with a diameter of 1 cm. Consequently, the measured toxin level offered insights into the ability of the examined *Pseudomonas* isolates to inhibit toxin production instead of their capacity to degrade toxins. The AFB1 content of the agar disk cut from the center of the axenic *A. flavus* control was 6.6 µg.

Remarkably, for 3 of the 50 *Pseudomonas* isolates (Ps-4, 66, 68), the AFB1 content in their interactions with *A. flavus* was less than 1% of the AFB1 levels observed in the axenic culture of the fungus (see Figure 1B). We discard the possibility that AFB1 production in these cases could match that of the axenic control, and that a bacterial degradative factor from the *Pseudomonas* isolates could diffuse effectively enough to completely eliminate the AFB1. Moreover, no AFB1-related compounds, such as precursors or degradation products were detected in these interactions, which rules out the possibility that a diffusible or volatile bacterial factor merely alters some steps in AFB1 biosynthesis or promotes its degradation. A more plausible explanation is that isolates Ps-4, Ps-66, and Ps-68 release a potent diffusible or volatile compound that directly inhibits the production of AFB1 in the fungus, possibly by interfering with key regulatory pathways.

In total, 22% of the isolates (*n* = 11) had no effect on the AFB1 content, while the remaining isolates (78%, *n* = 39) impacted the toxin level to varying degrees (Figure 1B). In type-A interactions, the levels of accumulated AFB1 were below 19.5%. In contrast, type-B and type-C interactions exhibited AFB1 contents of 9.1% (±0.6)–62.0% (±7.7) and 52.4% (±10.8)–103.0% (±6.8), respectively (Table S1). A correlation analysis revealed strong correlation (r = 0.88, *p* < 0.0001) between the toxin content and the type of interaction; however, no correlation was found between the isolates’ ability to control toxin production and their taxonomic classification (Figure 1B).

### 2.3. Effect of Pseudomonas Isolates on Fungal Growth and AFB1 Production in Liquid PD Medium (PDB)

The antagonistic potential of the isolated bacteria was also tested in liquid co-cultures. Based on fungal morphology and AFB1 accumulation, four types of interactions were distinguished. In type-I interaction, at least 39.3% suppression of *A. flavus* mycelial growth was observed, with the AFB1 content being undetectable; in type-II interaction, reduced mycelial development and reduced AFB1 accumulation compared to that of the axenic control was detected; in type-III interaction, reduced mycelial development and overproduction of AFB1 compared to that of the control was observed; and in type-IV interaction, no antagonistic effect was observed, neither on fungal growth nor on toxin production (Figure 1C). The AFB1 content of the fermented broth of the a xenic *A. flavus* control was 2.7 µg/mL. In total, 72% of the *Pseudomonas* isolates (*n* = 36) established type-I interactions, while 14%, 6%, and 8% of the isolates established type-II, -III, and IV interactions, respectively (Figure 1C).

Some *Pseudomonas* species have been demonstrated to be capable of degrading AFB1, presumably through enzymatic processes [31]. To test if our isolates possess the capacity to alter AFB1, we inoculated them into a PDB medium supplemented with 2 µg/mL AFB1. Subsequently, we measured the AFB1 content after 3 days of incubation. Given than no toxin degradation was observed with any of the isolates, we concluded that none of the fifty isolates degrade or transform the toxin in a PDB medium when bacteria are grown axenic.

The suppression of fungal mycelial growth to various extents might be attributed to the competition for nutrients or the presence of bacterial enzymes/metabolites that might influence the growth of mycelium.

Remarkably, we found substantial differences in AFB1 content when we compared the solid and liquid co-cultures of the same bacterial isolates. Specifically, the production of toxins by the fungus in type-III liquid interactions was enhanced upon interaction with isolates Ps-12, 31, and 84. However, these isolates exhibited type-B interactions on a solid medium; that is, they were able to reduce AFB1 production to 20–30% (with the control colony having 100% AFB1 content). The alteration of the AFB1-controlling capacity of Pseudomonads in solid and liquid co-cultures may be explained by either the generation of different solid-/liquid-phase-dependent bacterial effector compounds with opposing effects on fungal toxin production or by the different concentration of the same effector compound in liquid and solid media. The latter hypothesis is supported by studies reporting the overproduction of aflatoxin when *A. flavus* is exposed to suboptimal doses of antifungal agents [35]. Without elucidating the underlying mechanism, Campos-Avelar et al. also observed fungal toxin overproduction (ochratoxin) during the interaction of certain *Streptomyces* strains with *Penicillium verrucosum* [36].

The above results demonstrated that the antagonistic behavior of the bacteria is present to varying degrees in both liquid and solid co-culture; however, we did not find any significant correlation between data derived from solid and liquid cultures. Notable exceptions are three isolates (Ps-66, 68, 4) that completely inhibited the toxin production both in solid and liquid co-cultures.

### 2.4. Identification and Interpretation of the Formation of AFB1-Related Compounds

**Figure 2:**
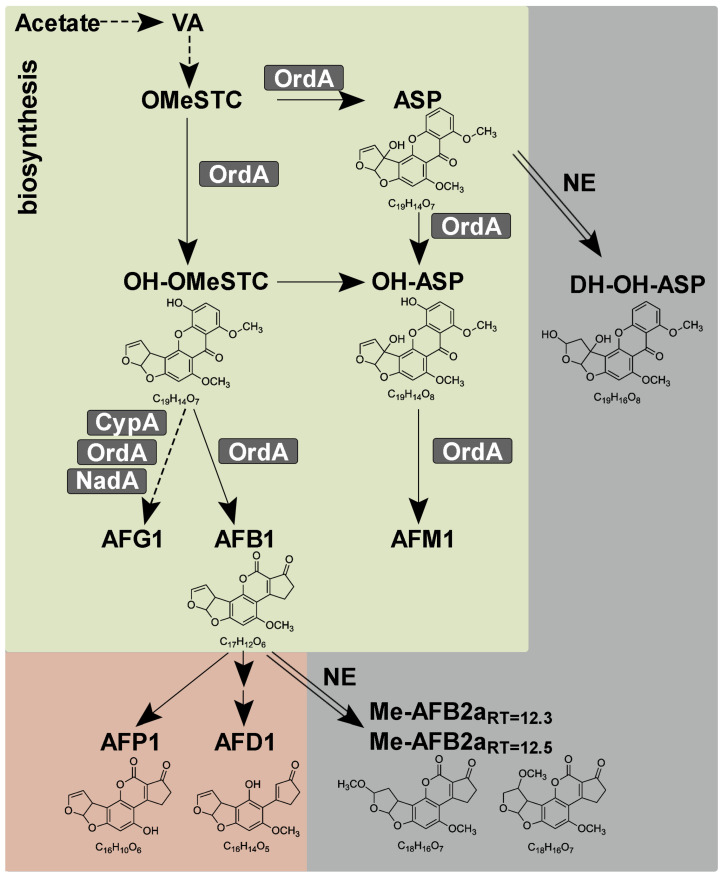
Biosynthetic pathway for aflatoxins (according to Udwary [37], and Yabe [38]) and downstream transformation reactions. Arrows and dashed arrows denote single and multiple enzyme reaction steps, respectively. Enzymes of reaction steps are indicated. Biosynthetic, enzymatic, and non-enzymatic degradation reactions are drawn with green, pink, and gray background colors, respectively. NE denotes non-enzymatic reactions (these steps are emphasized with double-line arrows). VA, versicolorin A; OMeSTC, O-methylsterigmatocystin; OH-OMeSTC, 11-hydroxy-O-methylsterigmatocystin; ASP, aspertoxin; OH-ASP, 11-hydroxyaspertoxin; DH-OH-ASP, dihydro-hydroxyaspertoxin; AFG1, AFB1, AFM1, aflatoxin G1, B1, M1.

Untargeted metabolomics studies, using high-performance liquid chromatography–high resolution mass spectrometric (HPLC-HRMS) analysis, were carried out in order to elucidate the underlying mechanism of the decreased AFB1 level in the interactions. AFB1 and its related compounds were identified from methanolic and chloroformic extracts of solid and liquid co-cultures, respectively. The identification of compounds that were structurally related to AFB1 was carried out by comparison of the experimental MS/MS fragmentation spectra of all compounds detected in the extracts to those of the toxin and its putative biotransformation-shifted fragments (oxidized, hydroxylated, dehydrated, etc.) (Figure 2, Table 1).

Amongst them, C_16_H_10_O_6_, C_16_H_14_O_5_, C_18_H_16_O_7_ (at two different retention times), and C_19_H_16_O_8_ could be considered degradation/transformation products, while C_19_H_14_O_7_ (at two different retention times) and C_19_H_14_O_8_ were precursors, i.e., known intermediates of the toxin biosynthetic pathway (Figure 2).

According to the literature, certain Aspergilli, including *A. flavus*, have the ability to degrade their own toxins [39,40] using their cytochrome P-450 monooxygenases [40], lactonases, and other reductases [41]. In our study, neither the used *A. flavus* strain in the applied media nor the *Pseudomonas* isolates could degrade or transform AFB1 when they were grown axenic (Figure 1, Table S2, and see above, respectively). The notion that AFB1-related compounds appeared only in certain interactions highlights that the co-cultured organisms could affect each other’s metabolic performances.

#### 2.4.1. Potential Role of Cytochrome P-450 Oxidoreductases and Other Oxidoreductases

Of the related compounds, C_16_H_10_O_6_ could correspond to AFP1 (aflatoxin P1), which might be formed by the demethylation of AFB1 (Figure 2). The MS/MS spectrum of this metabolite was in accordance with that published by Li et al. as a UV-light-induced transformation product of AFB1 [42] (Table 1). Although the cell-free extracts of *Trametes versicolor* had been reported to degrade AFB1 to AFP1, no MS/MS spectrum of AFP1 was provided to compare with our MS/MS spectrum [43].

The appearance of AFP1 during some interactions is explained by the enzymatic O-demethylation of AFB1. No AFB1 demethylase was identified in *A. flavus* so far; however, it is known that cytochrome P-450-type enzymes can catalyze this reaction in animals and humans [44]. In plant pathogenic *Fusarium* and *Nectria* species, a cytochrome P-450-type monooxygenase enzyme, pisatin demethylase (Pda1), was found to be responsible for the demethylation of the pisatin toxin produced by pea, whose compound structurally relates to aflatoxin [45]. Whether the demethylase contributing to the formation of AFP1 is of a bacterial or fungal origin remains an open question. The fact that AFP1 was only detected in solid co-cultures (Figure 1D) suggests that the fungal partner might be responsible for the conversion under limiting oxygen availability.

Two compounds with the same C_19_H_14_O_7_ formula, but with two retention times (RTs), and one compound with a C_19_H_14_O_8_ formula were 11-hydroxy-O-methylsterigmatocystin (OH-OMeSTC) or aspertoxin (ASP) and 11-hydroxyaspertoxin (OH-ASP), respectively (Figure 2). Their MS/MS spectra were in agreement with those previously reported [46] (Table 1). These metabolites can be considered the biosynthetic precursors of AFB1 and AFM1 (aflatoxin M1) [38]. In the biosynthesis pathway, the common precursor of both AFB1 and AFM1, O-methylsterigmatocystin (OMeSTC), is hydroxylated by the versatile OrdA enzyme (also known as AflQ, a cytochrome P450 family monooxygenase) at different carbons producing either ASP or OH-OMeSTC, which are further hydroxylated to OH-ASP (Figure 2) [38]. Both OH-OMeSTC and OH-ASP might be further transformed by OrdA, resulting in the production of AFB1 and AFM1, respectively [37,38] (Figure 2). AFM1 was not detected, neither in the axenic control culture nor in the co-cultures; however, ASP and OH-ASP precursors of this toxin and OH-OMeSTC were prevalent in the samples, especially ASP in the liquid co-cultures (Figure 1D).

Generally, intermediate compounds of a metabolic pathway could be detected when the pathway enzymes were either overproduced or became more active (e.g., due to the presence of accelerator compounds with low molecular weights) and the reaction substrate of the reaction with the slowest rate was accumulated. Therefore, it is feasible that increased OrdA overproduction or activity results in the observed accumulation of OH-OMeSTC, ASP, and OH-ASP compounds.

The detected C_16_H_14_O_5_ compound could be produced by the hydrolysis of the lactone ring, followed by decarboxylation (Figure 2). This compound is known as AFD1 (aflatoxin D1) and has been identified as a degradation product of AFB1 using a cell-free extract of *Escherichia coli* [47] or *Rhodococcus erythropolis* [48] and *P. putida* [30] cultures. Work by Zaccaria et al. revealed that AFD1 could be produced from AFB1 by laccases [49], although other enzymes, lactonases, and other non-defined enzymes are also considered to make this conversion [41]. As *A. flavus* can produce laccases [50], it is reasonable to suppose that this enzyme may contribute to the hydrolysis of the intramolecular ester bond (lactone ring) of AFB1 yielding AFD1. Remarkably, AFD1 was only detected in liquid interactions (Figure 1D). Whether the enzyme contributing to the formation of AFD1 is of a bacterial or fungal origin remains an open question.

The enhanced production or stimulation of oxidative enzymes by fungi upon fungal-bacterial interactions has been previously reported [51,52,53]. Based on this, it can be hypothesized that in response to certain antagonistic *Pseudomonas* bacteria, which are believed to release bioactive compounds, cytochrome P450 genes or other oxidoreductase genes in *A. flavus* are induced. This induction could lead to an elevated production of cognate enzymes, resulting in the formation of the above-described AFB1-related compounds.

#### 2.4.2. Compounds Produced by Non-Enzymatic Reactions

In the case of compound C_18_H_16_O_7_, there are two molecules eluting at different retention times, both possessing the same exact mass and fragmentation pattern (Figure 2, Table 1). The occurrence of C_18_H_16_O_7_ at RT = 12.3 min and RT = 12.5 min indicates that after the addition of a water molecule to the double bond of the furofuran ring, a methylether is formed (methoxy-aflatoxin B2a/Me-AFB2a) on one of the carbon atoms, leading to Me-AFB2a_RT=12.3_ and Me-AFB2a_RT=12.5_ (Table 1). The MS/MS fragmentation patterns of these compounds are in line with the results of Li et al. [42]. Hydration of the double bond of the furofuran ring (aflatoxin B2a or AFB2a) is a prerequisite for the formation of the Me-AFB2a compounds. It was shown that AFB2a is produced non-enzymatically in an acidic environment [54], followed by the further conversion of the hydroxyl group. Therefore, the emergence of Me-AFB2a compounds in the co-cultures could be interpreted as a result of acid accumulation. *A. flavus* is a known malic acid producer, which can be enhanced by decreasing the accessible nitrogen sources in the environment [55,56]. Therefore, it is feasible that a critical decrease in the nitrogen source concentration within the co-culture results in the accumulation of malic acid, which may trigger the formation of the methoxy derivatives of AFB2a.

An additional compound, C_19_H_16_O_8_, was also detected, which might be formed similarly to Me-AFB2a through the non-enzymatic addition of water to the double bond of the furofuran ring of OH-OMeSTC or ASP (Figure 2). By simulating and comparing the MS/MS fragmentation pattern of the two putative compounds with the experimental fragmentation patterns, we hypothesize that C_19_H_16_O_8_ might be the dihydrohydroxy derivative of aspertoxin (DH-OH-ASP) (Table 1).

#### 2.4.3. Occurrence of AFB1-Related Compounds in Interactions

No AFB1-related products could be detected in either solid or liquid interactions established by the *Pseudomonas* isolates, which belonged to interaction type-I (including all type-A isolates, 12 type-B, and 8 type C isolates) (altogether 36 isolates). We propose that the complete toxin biosynthetic pathway is blocked in these interactions. This is in line with prior transcriptomic studies, which revealed that certain bacteria [57] and fungi [58,59,60] are capable of inhibiting AFB1 production by downregulating the expression of the aflatoxin biosynthetic pathway genes through the production of bioactive factors.

AFB1-related compounds were identified exclusively in certain solid and liquid interactions (*n* = 12 and *n* = 11, respectively), in which AFB1 was produced (Figure 1D). Interaction with only four *Pseudomonas* isolates (Ps-7, 26, 31, and 84) resulted in the formation of AFB1-related compounds in both solid and liquid co-cultures. Co-cultures with other *Pseudomonas* isolates, listed in Figure 1D, resulted in the emergence of AFB1-related compounds either in solid or liquid interactions. Remarkably, the detection of AFB1-related compounds was not restricted to those interactions where antagonism was prevalent. These compounds were detected in type-III and certain type-IV interactions, where AFB1 was overproduced and the AFB1 content was comparable with that of the control, respectively (Figure 1D).

Among the AFB1-related compounds, AFP1 could only be detected in some solid co-cultures, whereas AFD1 was only present in some liquid co-cultures. All other compounds were identified in both solid and in liquid co-cultures in various quantities (Figure 1D).

Two toxin biosynthetic intermediates, ASP and OH-OMeSTC, were accumulated in all interactions, with two exceptions (ASP and OH-OMeSTC were missing from the solid interaction with Ps-84, and OH-OMeSTC was missing from the liquid interaction with Ps-24) (Figure 1D, Table S2).

The AFB1-related compounds in the tested samples showed an average of 7% and a maximum of 20% content relative to the AFB1 content of the axenic control colony within the solid co-cultures. The liquid co-cultures exhibited higher values than the solid ones: the samples showed an average of 18% and a maximum of 50% content relative to the AFB1 content of the axenic control. The increased proportion of the AFB1-related compounds in liquid co-cultures may be attributed to the enhanced exposure of the mycelia to the bacterial bioactive factor.

## 3. Conclusions

Due to the pressing need for sustainable biocontrol of *A. flavus*, extensive efforts have been made to identify more and more potent biocontrol agents and study the mechanism at the molecular level underlying the antagonistic effect of microbes. Here, we described three potent *A. flavus* antagonist *Pseudomonas* isolates (Ps-4, *P. paracarnis,* belonging to *P. fluorescens* species group, and Ps-66 and Ps-68 *P. chlororaphis* isolates, belonging to the *P. chlororaphis* species group), which completely blocked AFB1 production both in solid and liquid co-cultures, while only moderately reducing the fungal growth.

Given the potentially critical role of each species within a microbiome in maintaining diversity and balance among the member species of a local niche, the inability of the above three isolates to completely inhibit *A. flavus* growth may turn out to be a significant advantage for their application as biocontrol agents against *A. flavus* toxin production. Employing such agents is critically important, as they have the potential to both preserve microbiome diversity and diminish toxin production simultaneously. Since neither AFB1 nor any precursors or transformed derivatives of AFB1 were detected in the co-cultures, they supposedly act through releasing a diffusible or volatile compound, which blocks the operation of the whole toxin biosynthetic pathway. Given the diffusible or volatile nature of the bioactive compounds, these three *Pseudomonas* isolates might be excellent candidates as biocontrol agents for both open and closed environments, including various agricultural and storage settings.

In certain interactions, the detection and identification of AFB1-related compounds (precursors and transformation products of AFB1) suggest that a variety of fungal enzymes, including cytochrome P-450 oxidoreductases and laccases, might be overproduced or become more active in response to bacterial bioactive compound(s) and/or the accumulation of acids triggered by a critical decrease in available nitrogen sources. This observation indicates a complex interplay between bacterial presence and fungal metabolic responses, potentially leading to alterations in toxin production pathways.

## 4. Materials and Methods

### 4.1. Isolates and Media

*Pseudomonas* isolates were obtained from corn (*Zea mays*) rhizosphere soil from Madaras, Hungary (46°05′02.5″ N 19°15′46.5″ E), in 2021 by using *Pseudomonas*-selective media (10 g/L sucrose; 10 mL/L glycerol; 5 g/L casamino acids; 1 g/L NaHCO_3_; 1 g/L MgSO_4_; 2.3 g/L K_2_HPO_4_; 1.2 g SLS; 2% agar; 20 mg/L trimethropim) at 28 °C, followed by purification of the isolates on an LB medium (1% NaCl, 1% tryptone, 0.5% yeast extract). The purified monoclonal isolates were deposited at the Szeged Microbiological Strain Collection (SZMC) of the Department of Microbiology, University of Szeged, Hungary.

For species identification, isolates were grown in an LB medium overnight at 28 °C in the dark. Genomic DNA was extracted using the Zymo Research Quick DNA Fungal/Bacterial Miniprep Kit (Zymo Research, Irvine, CA, USA) according to the manufacturer’s instructions. Partial 16S and *rpoD* sequences were amplified using the primer sets Eub-8F/Eub-534R (5′-AGAGTTTGATCCTGGCTCAG-3′/5′-ATTACCGCGGCTGCTGG-3′) and PF1/PR1 (5′-GCGCAATCGCGCACTTCCC-3′/5′-GACATGCGACGGTTGATGTC-3′) and sequenced. Low-quality ends of the obtained sequences were removed, and all nucleotide positions were manually curated. The corrected sequences were submitted for similarity search using the NCBI nucleotide database (https://blast.ncbi.nlm.nih.gov/, URL accessed on 6 August 2024). The search was restricted to only include hits from type strains.

The AFB1 producer *A. flavus* was isolated from maize and deposited at SZMC (SZMC 21417, stored at −80 °C in 40% glycerol-supplemented YPD (1% peptone, 1% glucose, 0.5% yeast extract) medium, and used for the antagonism tests. The strain was grown on a PDA medium (VWR International, Radnor, PA, USA), and the conidiospores were collected and stored in 0.01% Tween-80 solution at 4 °C. During experimental work, fresh cultures were prepared by inoculation of the stored conidiospores to a PDA medium.

### 4.2. Antagonism Tests

For the solid co-cultures, Petri dishes with a 9.5 cm diameter filled with 50 mL of PDA medium were simultaneously inoculated with both *A. flavus* fresh conidiospore suspension (10 µL volume from a suspension with 10^5^ spores in 1 mL 0.01% Tween-80) and *Pseudomonas* isolates (20 µL of OD adjusted (OD_600_ = 0.8) cultures grown overnight at 28 °C with 130 rpm agitation in LB broth). The conidiospores were inoculated into the center of the plate, while the *Pseudomonas* isolates were inoculated in two spots flanking the centrally inoculated *A. flavus* at a 25 mm distance from the center. For the non-co-cultured (axenic) control, 10 µL of *A. flavus* fresh conidiospore suspension (10^5^ spores/1 mL 0.01% Tween-80) was spot-inoculated into the center of the PDA medium. The dishes were double-sealed with Parafilm and incubated at 28 °C for 8 days in darkness. Each experiment consisted of three technical replicates, and the experiments were performed at least in triplicate.

For the liquid co-cultures, 50 mL PD broth was simultaneously inoculated with 10 µL of *A. flavus* conidiospore suspension (10^5^ spores in 1 mL 0.01% Tween-80) and 40 µL of optical density (OD)-adjusted (OD_600_ = 0.8) culture of a *Pseudomonas* isolate grown overnight at 28 °C with 130 rpm agitation in an LB broth. For the axenic control, 10 µL of *A. flavus* conidiospore suspension (10^5^ spores/1 mL 0.01% Tween-80) was inoculated into 50 mL of PD broth. All cultures (co-cultures and axenic control) were incubated for 3 days at 28 °C with 130 rpm agitation. The experiments were carried out in triplicate.

### 4.3. Metabolite Extractions

Metabolites from the solid co-cultures and axenic control were extracted by removal of an agar block with a 1 cm diameter from the center of the fungal colonies by using a cork borer, followed by chopping up the agar blocks and adding 2 mL 80% (*v*/*v*) HPLC-grade methanol to them. To enhance the metabolite extraction, samples were sonicated with 80 kHz for 10 min at 25 °C and vortexed vigorously. After centrifugation (20 min at 4000× *g*), 1 mL of the supernatants was evaporated to dryness using a rotary evaporator (SpeedVac SC250EXPO, Thermo Fisher Scientific, Waltham, MA, USA). The samples were then stored at −20 °C until HPLC-HRMS analysis was conducted.

Metabolites from liquid co-cultures and axenic control were extracted by subjecting 1 mL of cell-free fermented broth (achieved by filtration) to repeated chloroform extractions (with equivalent volume). Chloroform phases were pooled and evaporated to dryness using a rotary evaporator (see above). Samples were then stored at −20 °C until HPLC-HRMS analysis was conducted.

### 4.4. HPLC-HRMS Analysis

The dried extracts were reconstituted in methanol, vortexed, and subjected to centrifugation at 19,000× *g* for 10 min. The resulting supernatants were subjected to HPLC-HRMS analysis.

HPLC-HRMS measurements were performed using a DionexUltimate 3000 UHPLC system (Dionex) coupled to an Q Exactive Plus hybrid quadrupole-Orbitrap mass spectrometer (Thermo Scientific, San Jose, CA, USA). In order to identify the analytes, a heated electrospray interface (HESI) was applied in both positive and negative ionization modes.

The AFB1-related compounds were separated using a Gemini-NX C18 (3 μm, 150 × 2 mm) (Phenomenex, Torrance, CA, USA) column. The mobile phases consisted of water (A) and acetonitrile/methanol (1/1) (B), both supplemented with 0.1% formic acid. A gradient elution profile was applied as follows: 0–2 min isocratic with 5% B; 2–13 min from 5 to 95% B; 13–19 min isocratic with 95% B; 19–19.5 min from 95 to 5% B; 19.5–24 min isocratic with 5% B. The flow rate was 0.2 mL/min, and the injection volume was 3 µL. The column temperature was maintained at 25 °C.

The ion source had the following settings: probe heater temperature 300 °C, ion transfer capillary temperature 320 °C, spray voltage 3.5 kV, sheath gas flow rate 30 arbitrary units, auxiliary gas flow rate 10 arbitrary units, and S-lens RF level 50 arbitrary units. The mass spectrometer data were acquired using a full-scan/data-dependent MS/MS method (Full MS/ddMS2). The full-scan MS spectra were acquired at a resolution of 70,000 from *m/z* 100 to 1200, with a maximum injection time of 100 ms. For every full scan, 10 ddMS2-scans were carried out with a resolution of 17,500 and a minimum automatic gain control target of 1.00 × 10^5^. The isolation window was 1.0 *m/z*. HPLC-HRMS data were acquired using Trace Finder 4.0 software. The raw MS data files were processed using Compound Discoverer™ (3.3) software.

AFB1 was separated using a Gemini-NX C18 (3 μm, 50 × 2 mm) column. The mobile phases consisted of water (A) and acetonitrile/methanol (1/1) (B), both supplemented with 0.1% formic acid. A gradient elution profile was applied as follows: 0–1 min isocratic with 5% B; 1–6 min from 5 to 95% B; 6–10 min isocratic with 95% B; 10–10.5 min from 95 to 5% B; 10.5–15 min isocratic with 5% B. The flow rate was 0.3 mL/min, and the injection volume was 3 µL. The column temperature was maintained at 25 °C.

The ion source had the following settings: probe heater temperature 350 °C, ion transfer capillary temperature 350 °C, spray voltage 3.5 kV, sheath gas flow rate 40 arbitrary units, auxiliary gas flow rate 10 arbitrary units, and S-lens RF level 50 arbitrary units. The mass spectrometer data were acquired using a parallel reaction monitoring method (PRM). Fragmentation was performed with a normalized collision energy of 50, and MS/MS scans were acquired at a resolution of 17,500 from *m/z* 100 to 500, with a maximum injection time of 100 ms and a target automatic gain control of 1.00 × 10^5^. The isolation window was 0.4 *m/z*. HPLC-HRMS data were acquired and processed using Trace Finder 4.0 software.

## Figures and Tables

**Table 1:** Mass spectrometric parameters of the detected AFB1-related compounds.

Molecular Formula	Putative Name	RT (min)	Precursor Ion		Product Ions (*m/z*)
			Form	*m/z*	
C_16_H_10_O_6_	AFP1	14.0	[M+H]^+^	299.0562	281.0451(53); 271.0604(32); 257.0446(82); 253.0495(21); 243.0653(13); 231.0290(100); 229.0498(58); 225.0544(12); 203.0342(22)
C_16_H_14_O_5_	AFD1	11.6	[M+H]^+^	287.0919	259.0619(16); 255.0651(100); 227.0704(19); 199.0759(12); 185.0593(11); 157.0657(19)
C_18_H_16_O_7_	Me-AFB2a	12.3	[M+H]^+^	345.0980	313.0718(100); 298.0489(2.6); 285.0763(58.2); 284.0684(13.7); 257.0807(9.2); 243.0654(2.8); 229.0855(2.3)
C_18_H_16_O_7_	Me-AFB2a	12.5	[M+H]^+^	345.0980	313.0712(100); 312.0630(2.6); 298.0484(2.9). 285.0762(61.3); 284.0685(14.5); 283.0610(2.7); 269.0442(2.0); 257.0807(9.9); 243.0652(2.6); 229.0856(2.7)
C_19_H_14_O_7_	OH-OMeSTC	12.2	[M+H]^+^	355.0828	340.0597(3); 327.0884(32); 299.0927(100); 285.0765(35); 266.0575(6); 255.0650(4)
C_19_H_14_O_7_	ASP	12.5	[M+H]^+^	355.0828	340.0596(75); 327.0885(8); 322.0491(100); 311.0564(16); 294.0532(7); 293.0453(4)
C_19_H_14_O_8_	OH-ASP	11.9	[M+H]^+^	371.0776	343.0833(44); 338.0440(16); 315.0876(100); 301.0719(38); 300.0636(22); 282.05316(20)
C_19_H_16_O_8_	DH-OH-ASP	10.7	[M+H]^+^	373.0933	337.0727(100); 327.0886(25); 313.0718(12); 309.0767(28); 297.0777(26); 295.0609(21); 285.0775(20); 281.0808(18); 271.0604(43); 267.0656(16); 255.0652(54)

AFP1: aflatoxin P1; AFD1: Aflatoxin D1; Me-AFB2a: aflatoxin B2a methyl ether; OH-OMeSTC: 11-hydroxy-O-methylsterigmatocystin; ASP: aspertoxin; OH-ASP: 11-hydroxyaspertoxin; DH-OH-ASP: dihydrohydroxyaspertoxin.

## Data Availability

The data presented in this study are available in this article and Supplementary Material.

## Supplementary Materials

The following supporting information can be downloaded at https://www.mdpi.com/article/10.3390/toxins16080367/s1: Table S1: AFB1 level and mycelium mass in the co-cultures; Table S2: Occurrence of AFB1-related compounds in the co-cultures.
