# Supplementary material for: Aflatoxin B1 Control by Various Pseudomonas Isolates
Source: Toxins (Basel). 2024 Aug 20;16(8):367. doi: 10.3390/toxins16080367 (PMC11358996; doi:10.3390/toxins16080367)
Supplement: Supplementary file 1 [file toxins-16-00367-s001.zip › toxins-3134572-supplementary.pdf]

Table S1. AFB1 level, and mycelium mass in the co-cultures.

	Strain	Level of AFB1 (% of control)	Level of AFB1 (% of control)	Mycelium mass (% of control)	Category	Category
		solid medium	liquid medium	liquid medium	solid medium	liquid medium
A. flavus	Control	100.7±5.6	100±6.6	100±4.6	Control	Control
P. putida	66	0.1±0	n.d.	12.6±2.2	A	I
P. putida	68	0.5±0.3	n.d.	13.7±2.3	A	I
P. putida	75	55.7±8.9	n.d.	31.9±5.6	B	I
P. putida	70	57.8±9.0	n.d.	24.9±4.1	B	I
P. putida	71	59.6±9.1	n.d.	27.7±4.5	B	I
P. fluorescens	4	0.9±0.3	n.d.	31.2±5.8	A	I
P. fluorescens	21	4.1±0.9	n.d.	9.2±0.9	A	I
P. fluorescens	9	7.6±0.8	n.d.	11.3±1.8	A	I
P. fluorescens	6	14.4±3.0	n.d.	21.0±3.0	B	I
P. fluorescens	27	17.8±3.2	n.d.	9.2±1.5	B	I
P. fluorescens	15	19.9±1.5	n.d.	12.3±2.1	B	I
P. fluorescens	12	20.0±2.3	187.3±25.7	67.5±10.6	B	III
P. fluorescens	31	27.8±4.6	138.4±15.4	62.9±10.1	B	III
P. fluorescens	7	32.7±4.5	55.7±7.2	57.3±8.0	B	II
P. fluorescens	20	39.0±2.0	n.d.	11.2±1.2	B	I
P. fluorescens	17	44.1±2.7	n.d.	17.3±1.9	B	I
P. fluorescens	29	44.9±6.4	42.1±6.1	48.3±7.5	B	II
P. fluorescens	76	45.8±7.8	65.8±9.1	51.8±8.2	B	II
P. fluorescens	74	50.0±4.2	n.d.	17.6±2.2	B	I
P. fluorescens	24	52.2±8.0	42.8±7.6	57.3±5.0	B	II
P. fluorescens	26	59.1±5.5	36.0±6.2	34.3±3.6	B	II
P. fluorescens	39	71.3±13.0	57.0±10.2	51.1±5.6	C	II
P. fluorescens	30	71.5±3.8	n.d.	19.2±2.5	C	I
P. fluorescens	44	77.9±11.3	64.4±10.3	49.7±4.2	C	II
P. fluorescens	8	79.3±10.0	n.d.	15.6±1.5	C	I
P. fluorescens	11	83.7±6.7	n.d.	33.9±5.0	C	I
P. fluorescens	1	84.7±13.9	n.d.	10.6±1.3	C	I
P. fluorescens	32	88.2±5.7	n.d.	27.7±3.7	C	I

	Strain	Level of AFB1 (% of control)	Level of AFB1 (% of control)	Mycelium mass (% of control)	Category	Category
		solid medium	liquid medium	liquid medium	solid medium	liquid medium
P. fluorescens	19	97.0±18.0	n.d.	23.6±4.4	C	I
P. jessenii	2	2.7±0.3	n.d.	13.4±1.9	A	I
P. jessenii	103	89.5±17.0	93.3±6.9	94.0±10.9	C	IV
P. koreensis	84	30.1±4.7	177.8±24.6	70.8±11.4	B	III
P. koreensis	99	37.8±1.5	89.4±14.2	83.2±7.1	B	IV
P. koreensis	101	53.5±12.0	96.9±12.7	91.0±7.6	B	IV
P. koreensis	72	55.9±12.2	n.d.	20.4±3.2	B	I
P. pohangensis	67	9.1±0.6	92.0±14.1	91.5±6.3	B	IV
P. putida	58	15.5±2.2	n.d.	39.3±5.1	A(F)	I
P. putida	59	15.6±1.7	n.d.	26.1±4.2	B	I
P. putida	60	17.0±2.1	n.d.	20.4±3.8	B	I
P. putida	57	19.5±3.5	n.d.	32.7±5.2	A(F)	I
P. putida	38	21.2±3.5	n.d.	21.8±3.7	B	I
P. putida	22	34.4±6.0	n.d.	8.0±0.8	B	I
P. putida	61	52.4±10.8	n.d.	9.1±1.6	C	I
P. putida	73	62.0±7.7	n.d.	9.5±1.6	B	I
P. putida	45	78.2±11.4	n.d.	19.4±3.6	C	I
unknown	89	93.7±13.8	n.d.	32.9±5.7	C	I
unknown	96	96.0±11.5	n.d.	37.2±6.1	C	I
unknown	90	101.0±9.3	n.d.	14.9±2.8	C	I
unknown	91	101.8±9.8	n.d.	15.6±2.6	C	I
unknown	100	103.0±6.8	n.d.	16.4±3.1	C	I

n.d. not detected

Table S2. Occurrence of AFB1-related compounds in the co-cultures.

	Type	Isolate	Amount (% of control)							
			AFP1	AFD1	Me-AFB2a (RT=12.3)	Me-AFB2a (RT=12.5)	OH-OMeSTC	ASP	OH-ASP	DH-OH-ASP
			C16H10O6	C16H14O5	C18H16O7	C18H16O7	C19H14O7	C19H14O7	C19H14O8	C19H16O8
SOLID MEDIUM	B	22	n.d.	n.d.	n.d.	n.d.	tr.	tr.	tr.	2.4±0.4
	B	84	9.9±1.6	n.d.	n.d.	n.d.	n.d.	n.d.	n.d.	n.d.
	B	7	6.8±1.3	n.d.	tr.	tr.	tr.	1.1±0.2	n.d.	n.d.
	B	15	n.d.	n.d.	1.7±0.3	1.8±0.3	tr.	tr.	n.d.	n.d.
	B	26	n.d.	n.d.	n.d.	n.d.	tr.	2.2±0.3	n.d.	n.d.
	B	31	1.3±0.2	n.d.	tr.	tr.	tr.	tr.	tr.	1.3±0.2
	B	60	tr.	n.d.	n.d.	n.d.	tr.	tr.	tr.	1.5±0.2
	C	1	3.2±0.5	n.d.	tr.	1.0±0.2	tr.	2.1±0.2	1.1±0.2	5.8±0.6
	C	8	tr.	n.d.	tr.	tr.	tr.	tr.	n.d.	n.d.
	C	11	n.d.	n.d.	tr.	tr.	tr.	tr.	n.d.	n.d.
	C	30	n.d.	n.d.	tr.	tr.	tr.	2.5±0.3	n.d.	n.d.
	C	32	3.5±0.3	n.d.	3.7±0.4	3.3±0.4	1.8±0.3	3.2±0.3	1.8±0.2	3.7±0.5
LIQUID MEDIUM	II	7	n.d.	n.d.	n.d.	n.d.	4.0±0.3	17.3±1.0	n.d.	n.d.
	II	24	n.d.	n.d.	n.d.	n.d.	n.d.	tr.	n.d.	n.d.
	II	29	n.d.	n.d.	tr.	tr.	tr.	tr.	n.d.	n.d.
	II	26	n.d.	n.d.	n.d.	n.d.	tr.	1.5±0.2	n.d.	n.d.
	III	31	n.d.	1.1±0.2	3.1±0.5	3.8±0.5	6.0±0.6	22.2±1.7	n.d.	n.d.
	III	12	n.d.	1.4±0.2	2.8±0.4	3.9±0.5	2.5±0.4	9.5±0.6	n.d.	n.d.
	III	84	n.d.	tr.	9.4±0.8	13.0±1	6.6±0.4	20.9±1.6	n.d.	n.d.
	IV	67	n.d.	n.d.	2.5±0.4	3.3±0.4	tr.	6.3±0.7	n.d.	n.d.
	IV	99	n.d.	n.d.	2.1±0.3	2.8±0.5	1.8±0.3	7.5±1.0	n.d.	n.d.
	IV	101	n.d.	1.1±0.2	2.2±0.3	2.6±0.4	3.3±0.3	13.7±1.4	n.d.	n.d.
	IV	103	n.d.	1.2±0.2	1.1±0.2	2.4±0.4	1.5±0.2	7.4±0.8	n.d.	n.d.

n.d. not detected

tr. less than 1% of control
